# Food for an Urban Planet: Challenges and Research Opportunities

**DOI:** 10.3389/fnut.2017.00073

**Published:** 2018-01-19

**Authors:** Dietrich Knorr, Chor San Heng Khoo, Mary Ann Augustin

**Affiliations:** ^1^Technische Universität Berlin, Berlin, Germany; ^2^North American Branch of the International Life Sciences Institute, Washington, DC, United States; ^3^Agriculture and Food, CSIRO Australia, Werribee, VIC, Australia

**Keywords:** food systems and urbanization, food challenge, food, nutrition, health and urbanization, food chain, food, nutrient, water security, sustainability, and safety, appropriate food production and processing

## Introduction

In 2014, Khoo and Knorr (1) identified the global shift in population demographics as one of the twenty-first century grand challenges, which warrants research prioritization by the food and nutrition communities. A persistent global growth trend toward urbanization can be attributed to this demographic shift. Satterthwaite et al. (2) characterized urbanization as “the increasing share of a nation’s population living in urban areas (and thus a declining share living in rural areas)” and stated that “Most urbanization is the result of net rural to urban migration, decline in rural population and gain in urban populations giving rise to megacities.” The global urban population is projected to increase by 1.84% per year between 2015 and 2020, by 1.63% between 2020 and 2025, and by 1.44% between 2025 and 2030. In 2016, 54% of the global population resided in urban areas; this is expected to rise to 60% by 2030, meaning that one in every three people will live in cities with more than half a million inhabitants. Meanwhile, the rural population is projected to decline from 45 to 40% between 2016 and 2030 (3) (Table 1). By 2050, more than 6.5 billion of the expected 9.6 billion global population will live in megacities with 10 million or more inhabitants, meaning that nearly two out of three individuals will be city dwellers (4–7).

**Table 1:** The growth in the world’s population by size of city, 2016 and 2030.

	2016			2030		
	Number of settlements	Population (millions)	Percentage of world population	Number of settlements	Population (millions)	Percentage of world population
Urban	–	4,034	54.5	–	5,058	60.0
10 million or more	31	500	6.8	41	730	8.7
5–10 million	45	308	4.2	63	434	5.2
1–5 million	436	861	11.6	558	1,128	13.4
500,000–1 million	551	380	5.1	731	509	6.0
Fewer than 500,000	–	1,985	26.8	–	2,257	26.8
Rural	–	3,371	45.5	–	3,367	40.0

*Adapted from the United Nations (3)*.

## Origin of Urbanization

The birth of human urbanization can be attributed in part to the discovery of fire and humans’ use of fire to provide warmth, protect from predators, and to cook food (8, 9). These new discoveries and technological advancements enabled humans to become more mobile and resourceful, as they traveled long distances in search of food and shelter. The discovery that cooking over fire made foods more palatable, appealing, digestible, and safer laid the foundation for modern-day food preparation, product innovation, and food distribution (10–13).

Improved postharvest preservation strategies, infrastructure, automation of farming, and distribution all enabled fresh produce to be produced on farms in regional areas and transported over long distances to urbanized areas that had a high population density but less land accessibility and capacity to produce food. This system of supplying food to dislocated urban areas from farms could have been sustainable if urban growth had been contained and at a slower pace. However, urban growth has been rapid, increasing from a population of 746 million in 1950 to 3.9 billion in 2014. Asia is home to 53% of the world’s urban population, followed by Europe (14%) and Latin America and the Caribbean (13%) (3). Both the farming community and the growing urban population will benefit from the development of industrial food processing hubs in agricultural regions, which are equipped with a range of unit operations capable of converting high volumes of agricultural produce into shelf stable ingredients and food products that can be transported to urban areas.

### Common Issues Associated with Urbanization

Understanding the common issues associated with urbanization will be critical to develop a better food supply model that is customized to large and smaller urban regions. First, urbanization is strongly correlated with economic growth and better standards of living. Countries that are the least urbanized tend to be among the poorest (14). Urbanization provides considerable advantages, including economic growth, centralized infrastructures, and government, political, education, social, and health services. This is evident in the long life expectancies seen in urban regions in European, Asian, and North and South American cities. However, urbanization has also resulted in many issues, such as urban poverty, food and nutrition insecurity, and health disparities (2, 15).

Second, food demands and dietary patterns in urbanized regions have shifted to more convenient and prepared foods, attributable in part to long working hours and better economic income among consumers (16, 17). Many of the convenient foods available in cities are also higher in salt, fats, and sugar than home-prepared foods. The available food choices are in part attributed to the change in eating patterns to favor a pattern high in saturated fat, salt, and sugar coupled with a sedentary lifestyle, restricted physical activity, close living proximity, and stress. These factors have contributed to increased risks of deaths from chronic diseases such as obesity, cardiovascular disease, diabetes, and renal failure.

Third, not all urban environments evolved similarly, and there is vast diversity in the characteristics of cities depending on their size (e.g., a small settlement with under half a million inhabitants or a large metropolis with over 10 million). Close to half of the world’s urban dwellers reside in relatively small settlements of fewer than 500,000 inhabitants, whereas only around one in eight live in the 28 megacities with more than 10 million inhabitants. By 2030, 41 megacities will surface in the Global South, and medium-size cities will be located in Asia and Africa (18).

Fourth, urbanization makes cities more susceptible to natural and human-made disasters (floods, droughts, and pollution). Climate change will affect food security and water access in areas that are already currently vulnerable (e.g., megacities) (19–21).

Fifth, societal trends and consumer preferences have driven the rise in peri-urban agriculture, as the urban population demands food from agricultural systems in close proximity to where they live. However, peri-urban land for agriculture has to compete with non-farming uses such as housing and regional industry, driving up the prices for land for agriculture in the city fringes (22). Land-use policies will dictate the trends for peri-urban agriculture. The search for alternative methods to access fresh produce in cities has resulted in the growth of urban agriculture, as seen in urban orchards in streets tended to by neighborhood communities, vertical farming on buildings, and rooftop gardens.

## Deciphering the Food System Challenges

### Accounting for a Resilient Food Supply System

**Figure 1:**
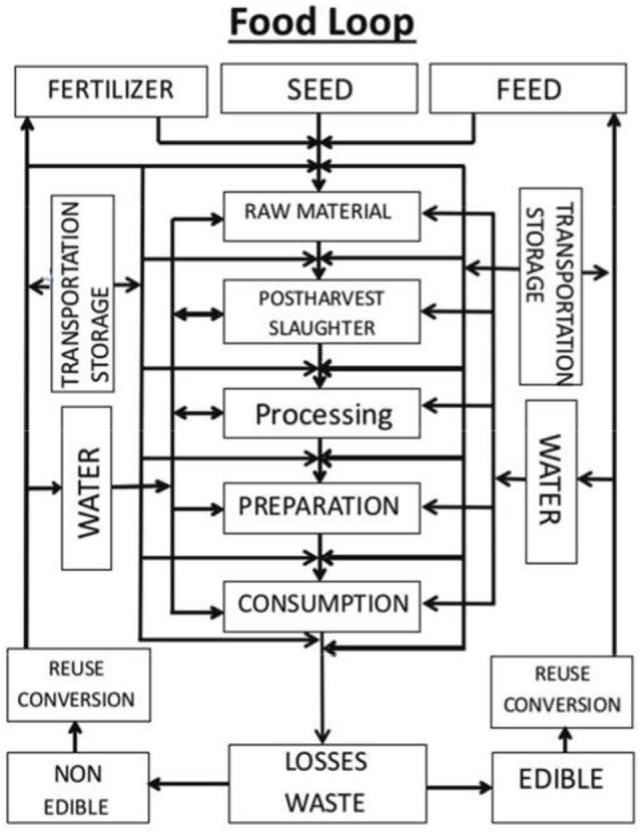
Proposed food loop for urban systems.

To ensure the sustainability and security of a viable food, water, and nutrition system for a growing urban population, many key factors influencing the food supply need to be addressed. These include food production, processing, distribution, preparation, and consumption (23). Figure 1 provides a simplified version of a “food loop” to illustrate the complexity and interconnectivity of the food system and recirculated food losses and wastes currently occurring in the food chain.

This complexity is compounded when other losses besides food losses, waste, and water utilization are added to the loop, including factors such as transportation, distribution, and storage issues that need to be considered at basically every step of the food chain. As a result, there is a need to plan for a more resilient urban food supply system. Such a system will need to reduce food loss and waste along the food chain, while at the same time promoting the growth of regional agricultural capabilities and the food industry (24). Recovering food loss from farm to retail that is otherwise lost to the food supply requires multipronged interventions including improved postharvest handling procedures, better data management and supply chain logistics, and the development of state-of the-art processing facilities for efficient conversion of edible underutilized biomass into stable food ingredients and consumer products. E-commerce operations will help create new pathways for supply of food to the consumers. However, equally important is to understand the drivers of consumer purchasing behavior in the urban population and the rising middle class and to involve consumers in a conversation about healthy food choices for a sustainable planet. In addition, the rapid rise in number, size, and diversity of megacities make the food chain and supply system, from field to tables, increasingly complex to manage. Managing these systems will require a Total System Approach incorporating multiple strategies including blockchain technology, Big Data analytics, computational modeling, the internet of things (IoT), and artificial intelligence (AI).

Because the conversion of farmland into urban areas is irreversible, it is important to integrate available digital technology (e.g., spatial digital mapping and soil quality analysis) to inform land-use policies (25). Larsen et al. (21) estimated that 10–40% of the global urban water supply is lost due to leaky pipes, with populations having access to improved water sources being especially low in areas of water scarcity. Apart from developing strategies to deal with issues in the earlier part of the food supply chain, there is also a need to engage with the consumer in the public conversation to reduce food loss and waste.

### Accounting for Impacts of Climate Changes

Besides production, processing, and packaging, the effects of climate change on agricultural production, population distribution, and urbanization need to be considered (26). Infrastructures in urban areas are usually more centralized, making them more susceptible to disruptions from flooding and power outages, which in turn affect food distribution, retailing, and food choices.

### Accounting for Dietary Changes

Most striking is the association between increased urbanization and increased away-from-home food consumption, reduced physical activity, and increased disease risk (7). The food industry needs to consider improving the range of health-promoting convenience foods to widen consumer choice for healthy eating in urban communities as well as providing more nutritious menus in restaurants and mobile catering trucks. The food processing, food service, and hospitality industries all have a role to play in accommodating the dietary choices of consumers and where they choose to eat. Through supplying to retail outlets and direct to consumers, the players in the food industry are striving to make available more affordable foods in health and wellness categories. These foods are delivered in various formats to cater for the behavior change associated with consumer food choices.

### Accounting for Food Safety

Considering the challenges discussed above as well as current existing food safety and transportation problems in megacities, sanitation and hygiene issues, water scarcity and pollution, food spoilage and wastes, risk of food poisoning, and impaired food transportation, storage, processing, preparation, and distribution are to be expected (27). Increased consumer awareness of food safety, chemical and microbial hazards, and digital transformation of food safety will help mitigate risks in the complex global supply chain. This also requires consideration of demographic shifts, regulatory policies, and consumer behavior (28).

### Accounting for Affordable Foods

It seems unlikely that current large-scale concentrated food production and processing will be adequate and affordable to catch up with the rapid and large-scale urban regional population influx. In some regions, small mobile markets have emerged in urban areas to provide more affordable and healthier food options to the under served in cities; however, these markets face significant political and economic challenges that need to be overcome (29).

## Research Needs to Address Food and Water Challenges

To address the multifaceted challenges discussed earlier, there is an urgent need to elevate the priority of these research areas in the food and nutrition communities. Transdisciplinary research that brings together multiple scientific research disciplines and the social sciences is necessary to facilitate the acceptance and adoption of new technology.

This section identifies several research needs to improve and advance current practices in urban food production and water management.

### Multidisciplinary Research

- Identify new strategies to develop food production activities within cities, including indoor and rooftop gardening, small land-farming, local urban garden sites (30), and microbial biomass or insects and products for food use (31, 32).
- While farming on local urban land has long been practiced and is acceptable to communities, producing food in and on urban buildings is newer, and the full implications of the latter have not yet been fully explored (33).
- Conduct studies to review and learn from successes and failures of ancient civilization food and water management practices. Barthel and Isendahl (34) also suggested studying food and water security among ancient civilizations such as the Mayans, who used integration of local production and subsequent storage of food and water in the boundaries of the city.
- Address new and emerging approaches and strategies for conserving water in an urban environment (21, 35). Such strategies should encompass the need to recover, recycle, and reuse urban water supplies. Water quality and consumption may be affected by the type of diets consumed (e.g., individuals who consume vegetarian diets may use fewer water resources than those who consume a meat-based diet) (36).
- Identify novel approaches learned from animal models such as insects (bees and ants). It may also be worthwhile to learn from the existing large-scale animal colonies and their organization including bees and ants.
- Develop smarter and more sustainable production systems. Advance crop research to provide more nutrient-dense crops to be grown in cities and to enable intake of more biofortified food to meet nutritional needs. There is a need and advantage to integrate policies of healthy dietary guidelines with sustainability guidelines (37, 38), including the integration of healthy sustainable diets with land appropriation for food.
- Integrate local rural and urban food production systems.
- Improve soil health for urban agriculture. Improved understanding of plant–microbiota–soil interactions and implications on crop quality, safety, and nutritional composition is needed.
- Explore new and alternative raw materials for the food supply chain and nutrient fortificants, including microbial biomass, plant biomass (cell/tissue/root cultures), single cell proteins, leaf protein concentrates, insects, and underexploited plant systems (39).
- Understand and improve urban food, nutrition, and health inequity, ending maldistribution of nutrition intake (40).
- Reduce biodiversity loss, water pollution, and unstable water withdrawal (41, 42).
- Identify new sources of water reservoirs and new concepts for water generation and recovery *via* bionics such as from desert plants and animals.
- Advance new strategies and technologies to reduce postharvest food losses.

### Advancing Food Processing and Distribution

- Create appropriate technology processes that are acceptable to the consumer and to industry.
- Develop urban food chain integration approaches and urban food system models including mass balance studies.
- Generate a worldwide inventory of appropriate, intermediate, or small-scale processing equipment and tools for knowledge and technology transfer and process development.
- Develop sustainable food urbanization concepts including zero-waste processes and emerging technology processes.
- Create food waste and water reduction and reuse models including low-energy processes such as fermentation, enzyme technologies, and membrane processes that take advantage of external energy sources such as solar energy.
- Build intracity transportation systems for food distribution and delivery.
- Integrate regional food production/processing systems with urban systems including urban process/preparation, such as street food and food vendors.
- Generate urban food, water, and waste recovery risk assessment models.
- Develop an integrated physical and virtual food loss bank to facilitate recovery of edible food loss for the food supply chain.

### Reducing Food and Consumption Waste

- Develop smart/intelligent packaging systems as integral food preparation/processing steps for away-from-home consumption.
- Develop smart interactive technologies such as in-home and wearable devices to inform and educate at industry and consumer levels the need and ways to reduce, recycle food waste.
  Involve consumers in a public conversation about responsible food purchase for healthy eating and reducing food waste as well as equipping consumers with intelligent decision-making tools.

### Assessing Public Policies on Food and Health

- Assess whether nutrition, dietary, and physical activity guidelines need to change with urbanization.
- Examine food and nutrition public policies and educational tools for an urban population, taking factors unique to urbanization into consideration (e.g., small and crowded spaces for food storage, production, and food safety in urban environments; food needs for the increased elderly population; nutritional, physical, and mental health issues related to city living; away-from-home and restaurant eating; and reduced physical activity). Integrate health policies with urbanized food system planning.

### Building Interdisciplinary and Transdisciplinary Approaches

- Elucidate and understand the basic mechanisms related to food systems and the importance of cross-disciplinary perspectives.
- Increase knowledge of food–human microbiota interactions and their implications on human health and future food and health innovation in an urban environment and couple this to personalized nutrition.
- Encourage multistakeholder engagement to promote a cocreated culture that facilitates transformational innovations, despite initial failures.
- Apply transdisciplinary approaches through strengthening interdisciplinary approaches with other disciplines of science and technology as well as with consumers, policy makers, and social scientists.
- Improve international knowledge and technology transfer regarding food science, other fields of science, and nature (bionics).
- Encourage a transdisciplinary and transpartisan dialog on the effects of urbanization on food and nutritional security in the face of climate change and depletion of natural resources.

## Discussion

The diversity of urban populations and the sizes of cities mean that the best strategies for managing food processing, food losses, and wastes are those that can be customized to a given region. This concept was introduced in the 1970s by Bates (43), but is still relevant today. For cities with limited infrastructure, customized processing facilities will be needed located within traffic and transportation limits of raw material supplies.

Unintentional food losses occurring mainly in developing nations and food waste in developed countries must be considered as well (44, 45). The development of a “food loss bank” has been suggested as a node in the future supply chain. This is predicted to be a networked physical and virtual stage-gated platform that will facilitate the diversion of safe, edible food loss biomass back into the food supply chain (46).

The need to rethink both the global supply chain and the inefficient use of storage has been addressed, and suggestions for improvement have been made (47). However, a holistic approach for intraurban transportation and storage has yet to be formulated.

## Conclusion

Urbanization is a result of modern living. Urbanization in conjunction with climate change will add new layers of complexity to the already existing grand challenges in food and nutrition (1, 48). Several recommended research areas and actions have been proposed in this paper. Tackling these challenges will require a concurrent intradisciplinary (within food science and technology), interdisciplinary (with other fields of science and technology), and transdisciplinary (with other fields of science and with society) approach to build agreement on strategies for and adoption of best practices for global sustainable food, nutrient, and water safety and security (49–52). Strategy development for true transformational innovation must take into account the interconnectedness of various risks (economic, environmental, geopolitical, and technological) (53) when forging new ideas to meet the challenges of ensuring food and nutritional security for the growing urban population.

## Author Contributions

All the authors contributed equally to this manuscript.

## Disclaimer

This paper reflects the opinions of Chor San Heng Khoo and may not be that of her affiliated institution.

## Conflict of Interest Statement

The authors declare that the research was conducted in the absence of any commercial or financial relationships that could be construed as a potential conflict of interest.
